# A Gentle Introduction to Lattice Field Theory

**DOI:** 10.3390/e27040341

**Published:** 2025-03-25

**Authors:** Erhard Seiler

**Affiliations:** Max-Planck-Institut für Physik (Werner-Heisenberg-Institut), Boltzmannstr. 8, 85748 Garching, Germany; ehs@mpp.mpg.de

**Keywords:** quantum field theory, lattice field theory, Quantum Chromodynamics

## Abstract

The principles of Lattice Field Theory (LFT), in particular Lattice Gauge Theory (LGT), are explained for a nonspecialist audience. We describe some of the successes of the program; we also discuss the relationship between LFT and Quantum Cellular Automata (QCA).

## 1. Introduction

Since Einstein and Minkowski, we are used to considering space–time as a continuum. But  nowadays this view is more and more being questioned [1,2,3]. Space–time is considered as “emergent” from something deeper, whatever that may be, or it is, due to quantum effects, assumed to have a “grainy” structure at the smallest distances comparable to the Planck length.

A much more mundane idea is to approximate the space–time continuum by a discrete structure, such as a lattice. This also has a long history: for example, there were the attempts by F. Bopp in the 1960s [4]; later there was the use of a lattice approximation in the enterprise of constructing Quantum Field Theories (QFTs) with mathematical rigor [5,6].

A pivotal event was the invention of Lattice Gauge Theory (LGT) by Ken Wilson [7], followed by the pioneering numerical study of a LGT by Michael Creutz [8]. Out of these beginnings developed a major industry: hundreds of physicists are working on extracting the physical implications of LGT with the aid of computing farms, sharing their results yearly in a major conference. The arXiv, the main repository of preprints in physics and related sciences, has had a section called hep-lat (for high-energy physics—lattice) since its inception in 1991. A number of good textbooks on LGT are also available, for example [9,10].

The main reason for the growth of LGT is that it made Quantum Chromodynamics (QCD), which describes the strong interaction of particles, also known as the nuclear force, amenable to numerical computation. Quantities like the masses of strongly interacting particles, called hadrons, which are not accessible by the usual perturbative treatment of QFT using Feynman graphs, could finally be computed. More about this in Section 7.

And then there is the approach to QFT featured in this volume, namely Quantum Walks and Quantum Cellular Automata (QCA) [11,12,13,14,15,16]. Of course, QCA are of great interest not only as discretizations of quantum systems but also as universal quantum computers or quantum Turing machines [17,18].

All these ideas and approaches share a central issue: they have to show the re-emergence of the continuum in the long wavelength limit, where the grainy or lattice nature at the microscopic scale should become invisible. We will discuss this for a simple case in Section 4 and briefly for QCD in Section 7.

## 2. From the Real to the Imaginary World

We are used to describing quantum systems by states (wave functions) evolving under unitary time evolutions; this is so also in QW and QCA. Typically, time evolution is generated by a self-adjoint, non-negative Hamiltonian

(1) $$H=H^{†}\;,\;H\geq 0\;.$$

*H* being nonnegative means that its spectrum is nonnegative. The ground state |0〉 has the lowest eigenvalue, which is taken to be 0 (if necessary by subtracting a constant from *H*).

The unitary time evolution is then given by

(2) U(t)≡exp(−iHt);

applying U(t) to a state ψ gives the solution of the Schrödinger equation

(3) $$\psi \left(t\right)=U\left(t\right)\psi \;,\;i\dot{\psi }\left(t\right)=H\psi \left(t\right)\;.$$

The first thing to notice is that U(t) can by analytically continued to complex times t−iτ, in particular negative imaginary time, a procedure also know as “Wick rotation” [19,20,21]. So, we replace *t* by −iτ and define

(4) K(τ)≡U(−iτ)=exp(−Hτ);

For τ≥0K(τ) is self-adjoint, bounded, and has spectrum between 0 and 1. It contains still all the information contained in U(t); in particular, the spectra (the eigenvalues) of *K* and *U* just differ by a factor *i*. *K* describes the evolution in the “imaginary world” but can also be used to describe finite temperatures state $\rho_{\beta }$ by identifying τ with the inverse absolute temperature β, and defining the thermal state by the density matrix

(5) $$\rho_{\beta }=\frac{1}{Z_{\beta }}K\left(\beta \right)\;,\;Z_{\beta }=\mathrm{tr}\;K\left(\beta \right)\;.$$

In particular, since the ground state has energy zero, we have

(6) K(τ)|0〉=|0〉

and

(7) $$\lim_{\tau \to \infty }K\left(\tau \right)=|0\rangle \langle 0|\;.$$

As is well known, the time evolution can be transferred from the wave functions to the operators (observables) via

(8) $$A\left(t\right)\equiv U\left(-t\right)AU\left(t\right)=U^{†}\left(t\right)AU\left(t\right)\;$$

(known as the Heisenberg picture); likewise, we can consider an imaginary time evolution via

(9) A(τ)≡K(−τ)AK(τ),

which implies

(10) $$A{\left(\tau \right)}^{†}=A^{†}\left(-\tau \right)\;,$$

where we use the notation

(11) $$A^{†}\left(-\tau \right)\equiv K\left(\tau \right)A^{†}K\left(-\tau \right)\;.$$

In quantum mechanics, the wave function is a function on the configuration space; for a nonrelativistic quantum particle, the configuration space, if simply the 3D space $R^{3}$ in which the particle moves with elements $\vec{x}$ and the wave function is a function $\psi (\vec{x})$. For two particles, the configuration space consists of pairs of vectors $\left({\vec{x}}_{1},{\vec{x}}_{2}\right)$, a wave function is a function $\psi ({\vec{x}}_{1},{\vec{x}}_{2})$, and so on for several particles.

In a (classical or quantum) field theory, the role of the configuration space is played by field configurations, which give the value of the field, such as the complex valued Higgs field of the Standard Model (SM) of elementary particles, for each space point. Denoting a generic field by ϕ, a field configuration is thus given by a function $\phi (\vec{x})$ and a wave function ψ is a function of a function, called a functional ψ[ϕ].

Feynman taught us that the unitary time evolution

(12) $$\exp(-iH\left(t_{2}-t_{1}\right))$$

has an integral kernel given by the “sum over paths”or “sum over histories”, weighted by a certain phase; symbolically, for a particle like an electron it is

(13) $$U\left(t_{1},t_{2};\vec{X},\vec{Y}\right)=\int_{\vec{X}\to \vec{Y}}D\vec{x}\left(t\right)\exp\left(iS\right)$$

where *S* is the action functional

(14) $$S=\int_{t_{1}}^{t_{2}}dtL\left[\vec{x}\left(t\right),\dot{\vec{x}}\left(t\right)\right]$$

and *L* is the Lagrangian corresponding to the Hamiltonian *H*. The integral $D\vec{x}\left(t\right)$ is over “all paths” $\vec{x}\left(t\right)$ moving from $\vec{X}$ to $\vec{Y}$ in the time interval from $t_{1}$ to $t_{2}$.

**Figure 1:**
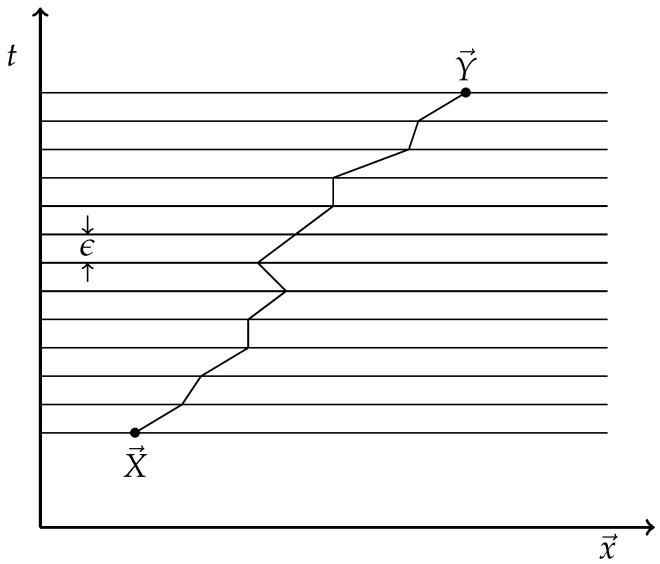
A piecewise linear path from ${\vec{x}}_{1}$ to ${\vec{x}}_{2}$.

To give the meaning of the mysterious integration symbol $D\vec{x}\left(t\right)$, Feynman and Hibbs [22] (see also [23]) approximate each path by a piecewise linear one, as  illustrated in Figure 1.

The path is cut up into small steps

(15) $$\{\vec{x}\equiv \vec{X},{\vec{x}}_{1},\ldots ,{\vec{x}}_{N}\equiv \vec{Y}\}\;,$$

happening in a small time interval ϵ. In each step from ${\vec{x}}_{j}$ to ${\vec{x}}_{j+1}$, the path is approximated by a straight line and “weighted” with a phase $\exp(i\epsilon S_{j,j+1})$, where $S_{j,j+1}$ the action for the linear piece from ${\vec{x}}_{j}$ to ${\vec{x}}_{j+1}$. We assume *L* to be a continuous function of $\vec{x},\;\dot{\vec{x}}$, let us say of the form

(16) $$L=\frac{{\dot{\vec{x}}}^{\;2}}{2m}-V\left(\vec{x}\right)\;.$$

To approximate $S_{j,j+1}$, we take for $\vec{x}$ midpoint ${\vec{X}}_{j,j+1}$ between ${\vec{x}}_{j}$ and ${\vec{x}}_{j+1}$; for $\dot{\vec{x}}$, we take the constant velocity on that linear piece. So, we approximate $S_{j,j+1}$ by

(17) $$S_{j,j+1}\approx \epsilon L\left({\vec{X}}_{j,j+1},\frac{{\vec{x}}_{j+1}-{\vec{x}}_{j}}{\epsilon }\right)\;.$$

The approximate expression for (13) using the piecewise linear path is then

(18) $$U\left(t_{1},t_{2};\vec{x},\vec{y}\right)\approx A{\left(\epsilon \right)}^{N-1}\int dx_{1}\ldots dx_{N-1}\exp\left(\sum_{j=1}^{N-1}\epsilon S_{j}\right)\;.$$

A(ϵ) is a normalization that has to chosen in such a way that the limit ϵ→0 exists. In the book [22], this limit is computed explicitly for various examples. Note also that $\sum_{j=1}^{N-1}\epsilon S_{j}$ is an approximation to the total action *S* (14).

The path integral does not really exist in the mathematical sense, but the mathematician Mark Kac [24] showed that using the imaginary time introduced above, the formal expression

(19) $$K\left(\tau_{1},\tau_{2};\vec{X},\vec{Y}\right)=\int_{\vec{X}\to \vec{Y}}D\vec{x}\left(\tau \right)\exp\left(-S\right)$$

where *S* is also analytically continued to imaginary time, can be given a precise sense, with the paths now in imaginary time.

If we are dealing with a relativistic quantum field theory, with a generic field denoted ϕ, the formula analogous to (19) is

(20) $$K\left(\tau_{1},\tau_{2};\Phi ,\Psi \right)=\int_{\Phi \to \Psi }D\left[\phi \right]\exp\left(-\int_{\tau_{1}}^{\tau_{2}}d\tau \int d^{3}xL\left[\phi \left(\tau ,\vec{x}\right),\dot{\phi }\left(\tau ,\vec{x}\right)\right]\right)\;,$$

where now L is the Lagrangian density. Here, the integral over D[ϕ] is the “sum of all histories” of the fields ϕ with $\phi \left(0,\vec{x}\right)=\Phi \left(\vec{x}\right)$ and $\phi \left(t,\vec{x}\right)=\Psi \left(\vec{x}\right)$. As before, the action functional is

(21) $$S\left[\phi \right]=\int_{\tau_{1}}^{\tau_{2}}d\tau \int d^{3}xL\left[\phi \left(\tau ,\vec{x}\right),\dot{\phi }\left(\tau ,\vec{x}\right)\right]$$

so that we have

(22) $$K\left(\tau_{1},\tau_{2};\Phi ,\Psi \right)=\int D\left[\phi \right]\exp\left(-S\left[\phi \right]\right)$$

In the imaginary world, the path integral is (typically) real, whereas in the real world it is complex. Moreover, in many cases in the imaginary world, the path integral is not only real, but exp(−S) is actually positive, so expressions like

(23) $$\langle A\rangle \equiv \frac{1}{Z}\int D\left[\phi \right]A\left[\phi \right]\exp\left(-S\right)\;;\;Z=\int D\left[\phi \right]\exp\left(-S\right)\;,$$

where A[ϕ] is some functional of the field ϕ, can be considered as expectation values in some probability distribution. This is the key to the numerical evaluation discussed in Section 6.

To make mathematical sense of (20) is not easy and so far has been achieved only in space dimension d<3; see [5,6]. It is important to note that in those cases it has been shown that the theory is invariant under the Euclidean group (rotations and translations) acting on the Euclidean space–time continuum. For this reason one also talks of the imaginary time version of QFT as Euclidean QFT.

## 3. Return to the Real World

As remarked, the imaginary world is also known as the Euclidean world, because in QFT it leads to a theory invariant under rotations and translations of the Euclidean space–time $R^{4}$. The Euclidean formalism had been used by Schwinger [21] long before it was known how to return from the Euclidean to the real (Minkowskian) world. This was achieved by Osterwalder and Schrader in the 1970s [25,26]. They formulated certain conditions on the Euclidean correlation functions (also known as Schwinger functions) that allowed the recovery of the Minkowski correlation functions (also known as Wightman functions) with all the right properties. The crucial property they identified is *Reflection Positivity* (RP).

RP is just an expression of the fact that Quantum Theory is based on a Hilbert space, i.e., a state space with a positive definite scalar product. In particular, the scalar product of a state (wave function) |ψ〉 with itself is positive:

(24) 〈ψ|ψ〉≥0.

If we take (see (9))

(25) |ψ(τ)〉=A(τ)|0〉

where |0〉 is the ground state (lowest energy state) of *H*, we find

(26) $$\langle \psi \left(\tau \right)|\psi \left(\tau \right)\rangle ={\langle 0|A\left(\tau \right)}^{†}A\left(\tau \right)|0\rangle =\langle 0|A^{†}\left(-\tau \right)A\left(\tau \right)|0\rangle \geq 0\;.$$

$A^{†}\left(-\tau \right)$ can be seen as the time reflected version of A(τ), hence the term “Reflection Positivity”.

In QFT, the fields become operators; we use the symbol φ to denote them. So, we now take for *A* a product of field operators and consider

(27) $$|\psi \rangle =|\varphi \left(\tau_{1},{\vec{x}}_{1}\right)\ldots \varphi \left(\tau_{n},{\vec{x}}_{n}\right)|0\rangle \;,\;\tau_{1}>\ldots >\tau_{n}\;,$$

where the ground state |0〉 is now usually known as the vacuum. We find, using $\varphi {\left(\tau ,\vec{x}\right)}^{†}=\varphi^{†}\left(-\tau ,\vec{x}\right)$

(28) $$\langle \psi |\psi \rangle =\langle 0|\varphi^{†}\left(-\tau_{n},{\vec{x}}_{n}\right)\ldots \varphi^{†}\left(-\tau_{1},{\vec{x}}_{1}\right)\varphi \left(\tau_{1},{\vec{x}}_{1}\right)\ldots \varphi \left(\tau_{n},{\vec{x}}_{n}\right)|0\rangle \geq 0\;.$$

Again, this is the vacuum expectation value of a time reflected operator times the unreflected one.

All these so far are obvious properties of the real quantum world. But, let us now express (28) by the Euclidean (imaginary time) functional integral:

(29) $$\frac{1}{Z}\int \exp\left(-S\right)\phi^{*}\left(-\tau_{n},{\vec{x}}_{n}\right)\ldots \phi^{*}\left(-\tau_{1},{\vec{x}}_{1}\right)\phi \left(\tau_{1},{\vec{x}}_{1}\right)\ldots \phi \left(\tau_{n},{\vec{x}}_{n}\right)D\left[\phi \right]\;.$$

As noted in (23) in the previous section, the expression (29) can be read as probabilistic expectation value 〈θ(A)A〉, where *A* is some functional involving fields at positive imaginary times τ and θ(A) is the time reflected and complex conjugate version of *A*.

An attentive reader may notice that in (28) the symbol $\varphi^{†}$ appears, whereas in (29), $\phi^{*}$ shows up. The reason behind this is that in (28), φ represents an operator, with $\varphi^{†}$ denoting the adjoint; in (29), ϕ is just a (complex) random variable and $\phi^{*}$ its complex conjugate. The two incarnations of the field are related in the following way: the probabilistic correlation functions of the random variables $\phi ,\phi^{*}$ are equal to the vacuum expectation values of the field operators $\varphi ,\varphi^{†}$ evolved to imaginary time:

(30) $$\frac{1}{Z}\int \exp\left(-S\right)\phi^{\#}\left(\tau_{1},{\vec{x}}_{1}\right)\ldots \phi^{\#}\left(\tau_{n},{\vec{x}}_{n}\right)D\left[\phi \right]=\langle 0|\varphi^{\#}\left(\tau_{1},{\vec{x}}_{1}\right)\ldots \varphi^{\#}\left(\tau_{n},{\vec{x}}_{n}\right)|0\rangle$$

where we assumed that $\tau_{1}<\ldots <\tau_{n}$; $\phi^{\#}$ stands for either ϕ or $\phi^{*}$, $\varphi^{\#}$ stands for either φ or $\varphi^{†}$.

We denote the set of functionals (actually a commutative algebra) living at positive imaginary times τ=−it as $A_{+}$. RP is then the statement

〈θ(A)A〉≥0foranyA∈A+.

RP allows us to create a Hilbert H space from $A_{+}$: first one takes the quotient space of $A_{+}/N$, where N is the *null space* of elements $A_{0}\in A_{+}$ which satisfy $\langle \theta \left(A_{0}\right)\;A_{0}\rangle =0$. This gives a so-called pre-Hilbert space, which can be completed to a Hilbert space:

(31) $$H=\bar{A_{+}/N}\;.$$

Quantum states (wave functions) are thus represented (non-uniquely) by elements of $A_{+}$. In particular, the unit element 1 represents the vacuum since it is invariant under imaginary time translations.

Translations in the positive τ direction by an amount σ

(32) τ↦τ+σ

produce matrix elements of

(33) exp(−σH)

as follows: let $A\left(0\right)\in A_{+}$, |ψ〉=A(0)|0〉 and A(σ) the functional obtained by replacing each τ by τ+σ; then,

(34) 〈θ(A(0))A(σ)〉=〈ψ|exp(−σH)|ψ〉.

By studying this type of expression for suitably chosen |ψ〉=A(0)|ψ〉, one can extract information about the spectrum of *H*, in particular masses of particles (see Section 5 and Section 6).

Everything so far is completely “formal”, in the sense of dealing with mathematically ill-defined quantities. In the next section, we introduce a space–time lattice to remedy this.

## 4. Lattice Field Theory

We now replace the space–time continuum by a simple hypercubic lattice $Z^{d+1}$. Some people might miss a lattice constant *a*, but as will be explained, this is not a parameter but rather a dynamically determined quantity and we work with the unit lattice until further notice.

The vertices of the lattice have coordinates

(35) $$n=(n_{0},\ldots ,n_{d})\;,$$

where bold-face symbols refer to (Euclidean) space–time vectors.

We think of $n_{0}$ as the discretized imaginary time, but in principle there is complete symmetry between the different coordinates.

A typical lattice action for a scalar field ϕ is

(36) $$S\left[\phi \right]=\frac{1}{2}\sum_{\langle nm\rangle }{\left(\phi \left(n\right)-\phi \left(m\right)\right)}^{2}+\frac{m_{L}^{2}}{2}\sum_{n}\phi {\left(n\right)}^{2}+\frac{\lambda }{4}\sum_{n}\phi {\left(n\right)}^{4}\;.$$

Here, the symbol 〈nm〉 denotes nearest neighbor pairs (links), oriented from n to m; the first two terms describe a free lattice field, whereas the the last term defines an interaction.

To make sense of the “sum over histories”, we first have to restrict ourselves to a finite piece Λ of the lattice and specify boundary conditions. Then, we integrate over all ϕ(n), n∈Λ, defining

(37) $$Z=\prod_{n\in \Lambda }\int d\phi \left(n\right)\exp\left(-S\left[\phi \right]\right)\;,$$

(38) $$\langle A\rangle =\frac{1}{Z_{\Lambda }}\prod_{n\in \Lambda }\int d\phi \left(n\right)\exp\left(-S\left[\phi \right]\right)A\left[\phi \right]\;,$$

where A[ϕ] is some functional, typically a polynomial of the fields. In the end, the thermodynamic limit $\Lambda \to Z^{d+1}$ has to be taken.

What we have defined here can be considered as a system of classical statistical mechanics (CSM); so, all the techniques of CSM, like cluster expansions and concepts like phase transitions and critical phenomena of CSM can be applied.

LGT≅CSM

Luckily, with the action (36) RP holds (see [27,28,29]), provided the lattice is symmetric under time reflections. If the infinite volume limit in time direction has been taken, and the spatial extent of Λ does not depend on the time coordinate, we can also introduce the time shift introduced before (albeit only by integer amount); shifting by one lattice spacing in imginary time, we obtain an operator T to be interpreted as

(39) T=exp(−H).

T is known in CSM as the *transfer matrix*.

Let us return to the issue of the lattice constant. If we look at the case λ=0 in (36), all the integrals are Gaussian and can be evaluated explicitly. We find, for instance (after taking the infinite volume limit),

(40) $$G\left(n-m\right)\equiv \langle \phi \left(n\right)\phi \left(m\right)\rangle =\frac{1}{{\left(2\pi \right)}^{d+1}}\prod_{\nu =0}^{d}\int_{-\pi }^{\pi }dp_{\nu }\frac{\exp(ip\cdot (n-m))}{\sum_{\nu =0}^{d}\left(2-2\cos p_{\nu }\right)+m_{L}^{2}}\;.$$

This expression decays exponentially for large distances |n−m|:

(41) $$\langle \phi \left(n\right)\phi \left(m\right)\rangle ∼\exp(-|n-m|/\xi_{L})$$

with $\xi_{L}=O\left(1/m_{L}\right)$ for small $m_{L}$. $\xi_{L}$ is the so-called correlation length in lattice units. It should be used to ‘calibrate’ the lattice by using it to define the standard of length. For instance, we may declare $\xi_{L}$ to represent the physical length ξ=1.0 fermi (fm); in this new incarnation, we denote it by ξ without a subscript, which now has the dimension of length.

If, for instance, $\xi_{L}=10$ in lattice units, the lattice constant in physical units is

(42) $$a=\frac{\xi }{\xi_{L}}=\frac{1.}{10}=0.1\left[\mathrm{fm}\right]\;.$$

A vector n in lattice units thus corresponds to a vector

(43) x=na

with components measured in fermi. Likewise, a wave vector p as appearing in Equation (40) corresponds to a momentum vector

(44) k=p/a

and the mass parameter $m_{L}$ corresponds to a mass

(45) $$M=m_{L}/a$$

in physical units. Here, we used a convention that is standard in particle physics: we set the speed of light and the reduced Planck constant *ℏ* equal to 1, and the mass *M* then has indeed dimension 1/length, as shown in (45). One fm then corresponds to a mass of 1.2398 GeV, which is of the order of the baryon masses.

We have obtained quantities carrying a dimension from the dimensionless lattice quantities by the simple declaration making ξ a physical length. This is an instance of known as *dimensional transmutation*.

The continuum limit is obtained by sending the lattice constant to 0, or equivalently the correlation length to *∞* and the the mass $m_{L}$ to zero. In the language of statistical mechanics, one is going toward a critical point. By rescaling and renormalizing G(n−m) in accordance with (43)–(45) we obtain the renormalized two-point function

(46) $$G_{r}\left(x-y\right)\equiv Z{\left(a\right)}^{-1}G\left([x-y/a\right])$$

with $Z\left(a\right)\propto a^{d-1}$ for d>1 (in d=1 one has to take Z(a)∝loga). It is easy to see that the limit a→0 of $G_{r}$ exists and is

(47) $$\frac{1}{{\left(2\pi \right)}^{d+1}}\int \frac{\exp((x-y)\cdot k)}{k^{2}+M^{2}}d^{d+1}k$$

(for the mathematically scrupulous, the integral is to be interpreted as the Fourier trasnformation of a distribution).

In the continuum limit, we find the simple relation

(48) $$\xi =\frac{1}{M}\;,$$

which is the well-known relation between mass and Compton wavelength.

The same principle is used for the interacting case λ≠0; the correlation length cannot be determined explicitly in this case, but it can be computed by numerically simulating the system (see Section 6). The continuum limit will be discussed in a more general setting in Section 7.

## 5. Lattice Gauge Theory

Lattice Gauge Theory, in its general form, was invented in 1974 by Ken Wilson [7]; a special case of it (the gauge invariant Ising model) had been introduced earlier by Franz Wegner [30].

The idea of gauge transformations and gauge invariance originates in the classical theory of electromagnetism, where the vector potential is determined by the electromagnetic field only “up to gauge transformations”. H. Weyl first introduced the term “gauge” in 1918 in an (unsuccessful) attempt to unify Einstein’s General Relativity with electromagnetism, but in 1929 he successfully introduced the concept in quantum mechanics, where a gauge transformation refers to space dependent shift of the phase of the wave function combined with a related transformation of the electromagnetic vector potential. This is based on the underlying group of transformations U(1) (see below).

The next step was the introduction of non-abelian (non-commutative) generalization by C. N. Yang and R. L. Mills in 1954 [31], formulating what is known today as Yang–Mills theory. This concept is an essential ingredient of the *Standard Model* (*SM*) of particle physics, which describes—so far perfectly—the strong, weak and electromagnetic interactions of so-called elementary particles, including electrons, muons and quarks.

The gauge group of the SM is rather complicated: it is the direct product of the groups U(1),SU(2),SU(3). Here

(49) $$U\left(1\right)=\left\{e^{i\alpha }|0\leq \alpha <2\pi \right\}\;$$

is the group of rotations in the plane, which is abelian (=commutative); SU(N) is the non-abelian group of unitary N×N matrices with determinant 1.

Instead of explaining the original continuum version of gauge field theories, I will immediately go to Lattice Gauge Theory (LGT), which is easier to describe anyway. I will focus on the simplest non-abelian group SU(2).

The elements of SU(2) can be written es

(50) $$U=\left(\begin{array}{cc} a_{0}+ia_{3} & ia_{1}+a_{2}\\ ia_{1}-a_{2} & a_{0}-ia_{3}\;, \end{array}\right)$$

where the $a_{i}$ are real numbers satisfying

(51) $$a_{0}^{2}+a_{1}^{2}+a_{2}^{2}+a_{3}^{2}=1\;.\;$$

so they are describing the unit sphere in four dimensions.

**Figure 2:**
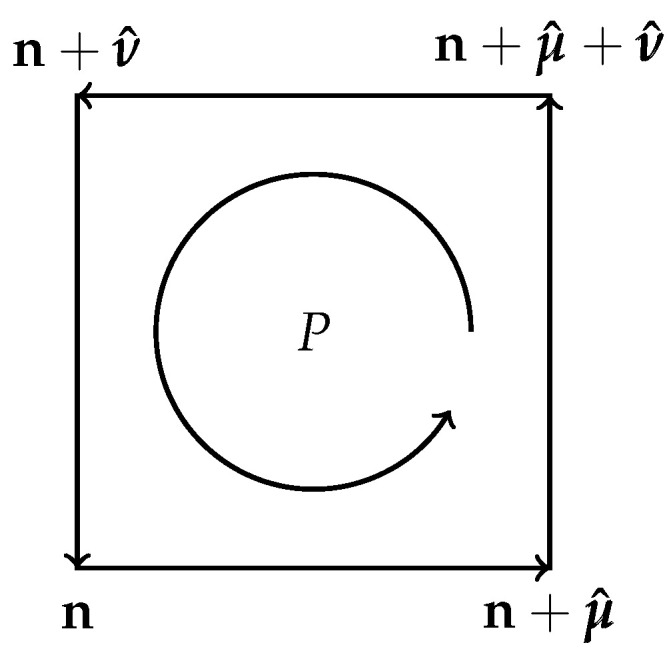
An oriented plaquette.

To define LGT, we need, in addition to the vertices and links (nearest neighbor pairs) encountered in the previous section, the so-called plaquettes of the lattice: a plaquette is an oriented elementary square of the lattice, i.e., the simplest closed loop made from for links (see Figure 2). Denoting by $\hat{\mu }$ the unit vector on the lattice in direction μ, we define a lattice gauge field as a map from the links into the gauge group:

(52) $$\langle n,n+\hat{\mu }\rangle \mapsto U_{\mu }\left(n\right)$$

with the property

(53) $$\langle n+\hat{\mu },n\rangle \mapsto U_{\mu }{\left(n\right)}^{-1}=U_{\mu }{\left(n\right)}^{†}\;,$$

i.e., reversing the direction of a link requires taking the inverse of the gauge field *U*.

**Gauge field action:** The simplest action is *Wilson’s action* (there are other choices):

(54) SW[U]=2g02∑PRetrUP,

where the sum is over all the plaquettes *P* and

(55) $$U_{P}=U_{\mu }\left(n\right)U_{\nu }\left(n+\hat{\mu }\right)U_{\mu }{\left(n+\hat{\nu }\right)}^{-1}U_{\nu }{\left(n\right)}^{-1}$$

and $g_{0}^{2}$ is the (bare) coupling constant. In a naive continuum limit, this expression corresponds to the action density $\mathrm{tr}F_{\mu \nu }^{2}$, in close analogy to the well-known action of the electromagnetic field.

**Gauge Invariance:** The action (54) is invariant under the transformation of the gauge field

(56) $$U_{\mu }\left(n\right)\mapsto V{\left(n\right)}^{-1}U_{\mu }\left(n\right)V\left(n+\hat{\mu }\right)\;,$$

where V(n) is any assignment of a unitary matrix to the vertices n.

The gauge field can be coupled to matter fields living at the vertices of the lattice:

**Scalar matter field:** We need a scalar lattice field which has several components, such that the gauge transformations (56) can act (mathematically speaking, the scalar field has to allow a representation of the gauge group). The simplest choice for the case of SU(2) is a two-component complex field ϕ (actually, the right choice for the Higgs field of the SM).

A gauge-invariant action for a scalar field interacting with the gauge field is

(57) $$S\left[\phi ,U\right]=\frac{1}{2}\sum_{n,\mu }{\left(\phi \left(n\right)-U_{\mu }\left(n\right)\phi \left(n+\hat{\mu }\right)\right)}^{2}+\frac{m_{0}^{2}}{2}\sum_{n}\phi {\left(n\right)}^{2}+\frac{\lambda }{4}\sum_{n}{\left(\phi {\left(n\right)}^{2}\right)}^{2}+S_{W}\left[U\right]\;,$$

where a gauge transformation V(n) acts on the scalar field as

(58) ϕ(n)↦V(n)ϕ(n).

**Fermionic matter field:** We denote a fermionic field by $\psi_{\alpha }\left(n\right)$, indicating that there are several components for each lattice site n. The fields anticommute and are nilpotent, forming a so-called Grassmann algebra.

For the lattice functional integral we need two more prescriptions:

(a) The integration over the fermion field ψ is the so-called Berezin integral, ref. [32] which is defined by the rule

(59) $$\int d\psi_{\alpha }\left(n\right)=0\;,\;\int \psi_{\alpha }\left(n\right)d\psi_{\alpha }\left(n\right)=1\;$$

together with the anticommutation relations

(60) $$\left\{\psi_{\alpha },\psi_{\beta }\right\}=\left\{d\psi_{\alpha },d\psi_{\beta }\right\}=0\;\forall \alpha ,\beta \;.$$

(b) The integration over the lattice gauge field has to be performed with an invariant measure (Haar measure); for the case of SU(2), this is simply the rotation invariant measure on the three-sphere defined by (51).

A typical form of the action for a LGT with gauge and fermion fields, such as Quantum Chromodynamics (QCD) is

(61) $$S=S_{f}+S_{W}\;,$$

where the fermionic action is, for instance,

(62) $$S_{f}=\sum_{n,\alpha }{\bar{\psi }}_{\alpha }\left(n\right){\left(D_{W}+m_{0}\right)}_{\alpha \beta }\psi_{\beta }\left(n\right)\;.$$

Here, ${\bar{\psi }}_{\alpha }$ is another fermion field, independent of $\psi_{\alpha }$, and $D_{W}$ is a lattice version of the Dirac operator, made *gauge covariant* by the coupling to the gauge field. The version proposed by Wilson [7] is:

(63) $$D_{W}=\frac{1}{2}\left(\gamma_{\mu }\left(D_{\mu }^{*}+D_{\mu }\right)-D_{\mu }^{*}D_{\mu }\right)\;;$$

the $\gamma_{\mu }$ are the Dirac matrices and $D_{\mu }$ is the covariant difference operator

(64) $$D_{\mu }\psi \left(n\right)=U{\left(n\right)}_{\mu }\psi \left(n+\hat{\mu }\right)-\psi \left(n\right)\;.$$

Wilson proposed (63), which contains the naively unexpected second order difference operator in order to solve the so-called fermion doubling problem that would arise without that term [9,10,33]. A detailed discussion of this issue is beyond the scope of this introduction. Some idea of the complexity of this issue can be gained by consulting the Wikipedia article on fermion doubling [34].

In the SM, the fermion fields describe the leptons and quarks. They are multicomponent fields with the gauge group acting unitarily on the indices, e.g.,

(65) $${\left(V{\left(n\right)}_{\mu }\psi \left(n\right)\right)}_{\alpha }={\left(V{\left(n\right)}_{\mu }\right)}_{\alpha ,\beta }\psi {\left(n\right)}_{\beta }\;.$$

## 6. Numerical Evaluation of Lattice Gauge Theory

The Berezin integral is not well suited for direct numerical evaluation, but the actions used in the Standard Model are always quadratic in the fermion fields and therefore these fields can be “integrated out” using the general formulae like

(66) $$\prod_{k}\int d{\bar{\psi }}_{k}d\psi_{k}\exp\left(\sum_{j,k}{\bar{\psi }}_{j},A_{jk}\psi_{k}\right)=\det\left(A\right)\;,$$

where the indices j,k now stand for both the lattice points n and the components of the fermion field. A derivation of this formula is given in the Appendix A.

A “lucky coincidence” is the fact that the determinant appearing in (66) is generally positive (exceptions are field theories with a nonzero chemical potential for the fermions and those with a so-called topological term, which pose a serious difficulty for numerics, the so-called sign problem). This positivity makes it possible to evaluate the functional integrals via “importance sampling”, such as the Monte Carlo method. Nowadays, refined versions of this are in use, for instance, for instance something called “hybrid Monte Carlo” (HMC).

Generically, the idea is to define an algorithm that produces stochastically a sequence of field configurations; on each configuration, a number of quantities (“observables”) are evaluated. The algorithm is set up in such a way that in the limit of infinitely long sequences the averages of the observables converge to their expectation values in the functional integral. For a detailed description on how this works, I recommend the textbooks mentioned before [9,10].

Let me now give a rough sketch of how particle masses are extracted from LGT, at first in unphysical lattice units. First of all, since

(67) $$\lim_{t\to \infty }\exp\left(-tH\right)=|0\rangle \langle 0|\;$$

where the right hand side is the projection operator onto the ground state of *H*, the true ground state will emerge in the infinite volume limit automatically; remember that it is represented by the function 1 in the functional integral.

Physical states representing particles are obtained by inserting functionals (operators) with the appropriate quantum numbers. In LGT, these functionals have to be chosen gauge invariant, unless we want to represent some unphysical infinitely heavy external charges. By looking at the exponential decay rate of the two-point correlator of such operators, we obtain the lowest energy (=mass) among the states with the quantum numbers chosen, as described in Section 4.

Let us, for instance, consider the η meson in a simplified version of QCD, in which only the two lightest quarks (“up” and “down”) are present. It is a pseudoscalar meson with isospin 0, so an operator with the right quantum numbers is

(68) $$\chi_{\eta }\left(x\right)\equiv {\bar{\psi }}_{up}\left(n\right)\psi_{up}\left(n\right)+{\bar{\psi }}_{down}\left(n\right)\psi_{down}\left(n\right)\;.$$

$\chi_{\eta }\left(x\right)$ is obviously gauge invariant. We consider the correlation function

(69) $$\langle \chi_{\eta }\left(n\right)\chi_{\eta }\left(n+k\;\hat{0}\right)\rangle \;,$$

where $\hat{0}$ is the unit vector in the time direction. This function will decay exponentially for large lattice distances, as long as we are not at a critical point. The exponential decay rate will in general depend on the quantum numbers used; since we chose those of the η meson, we interpret the asymptotic behavior as $\exp(-m_{L}\left(\eta \right)k)$, where $m_{\eta }$ is the mass of the η meson, so far in the unphysical lattice units. To obtain such masses in physical units, we have to calibrate the lattice spacing in physical units. How this is accomplished was described in Section 4 for a free field and we will return to it in the next section.

Of course, there are more clever choices for the operator $\chi_{\eta }$; the first improvement is to take the sum over the spatial part of the lattice, i.e., fix the momentum to 0.

## 7. Renormalization and Continuum Limit

We have described in some detail in Section 4 how the continuum limit is constructed for the trivial case of a free scalar field. We now sketch how this is accomplished in the context of a more complicated theory, such as lattice QCD.

In this case, there is more than one correlation length, depending on the quantum numbers of the observables whose correlations we consider. As in the trivial free case, these correlation lengths are proportional to the inverse masses (in lattice units) of the particle appearing in this channel. One of these masses has to be picked to define the reference mass $M_{r}$ (which corresponds to the inverse of a standard of length as described in Section 4) by equating it with the corresponding experimentally measured mass. Again, by this choice, we produce a *dimensional transmutation* that gives dimension to the lattice parameters.

The first step of renormalization is expressing of all lengths and masses in physical units by using the reference mass $M_{r}$ (or the corresponding correlation length) as the standard.

After this trivial renormalization has been performed, one can try to take the continuum (=long wavelength) limit. Taking the continuum limit of a lattice field theory is necessary even if one does not believe in the reality of the continuum. This is so because in the absence of any detailed knowledge about the possible breakdown of the continuum at short distances, one still wants to extract those aspects of the theory that do not depend on the unknown features of that breakdown.

A QFT is completely determined by its Euclidean or Minkowskian correlation functions. The continuum limit for these correlation functions of LFT/LGT requires the following: we first renormalize all masses and lengths as stated above, and then we vary the parameters of the theory in such a way that the reference scale grows to infinity in lattice units. As explained in Section 4, this means that we have approach a *critical point* of the model in question; in a theory like QCD, the critical points actually form a manifold and a priori any point on that manifold determines a continuum limit.

To describe renormalization and the continuum limit for the correlation functions, we have to renormalize also the field strength (cf. Section 4). Let us consider a scalar field theory to keep the notation simple. So, let

(70) $$G_{lat}\left(n_{1},\ldots ,n_{N}\right)\equiv \langle \phi \left(n_{1}\right)\ldots \phi \left(n_{N}\right)\rangle$$

be the correlation function of *N* fields on the unit lattice. The renormalized correlation function on the lattice with lattice constant $a\propto 1/\xi_{L}$ (where $\xi_{L}$ is the correlation length chosen to fix the length standard) is then

(71) $$G_{ren}\left(x_{1},\ldots ,x_{N}\right)=Z{\left(a\right)}^{-N/2}G\left(\left[x_{1}/a\right],\ldots ,\left[x_{N}/a\right]\right)\;,$$

where [x/a] is the integer part of x/a. Then, the continuum correlation functions should be defined as

(72) $$G_{cont}\left(x_{1},\ldots ,x_{N}\right)=\lim_{a\to 0}Z{\left(a\right)}^{-N/2}G\left(\left[x_{1}/a\right],\ldots ,\left[x_{N}/a\right]\right)\;.$$

Z(a) (“wave function renormalization”) is a factor that has to go to zero as a→0 in such a way that the limit exists and is nontrivial (as discussed in detail in Section 4 for a free scalar field). If everything works out, Z(a) can be chosen independent of the number *N* of fields and the limit satisfies a number of properties; in particular, it is invariant under rotations. As remarked earlier, this is not easy and has so far only be achieved in space–time dimensions less than four. The continuum limit will always inherit RP from its lattice approximation.

In the numerical evaluation of a LGT, such as Lattice QCD, one is not so much interested in correlation functions; rather, one wants to extract the masses of the hadrons (protons, neutrons, hyperons, pions, kaons etc.).

QCD has a number of free parameters, such as bare quark masses. The critical manifold is parameterized by some of the parameters of the theory. One has to use a number of experimental inputs to fix the point on the critical manifold one wants to approach, but the number of inputs is much smaller than the number of masses predicted by lattice QCD. So, comparison of theory and experiment is quite stringent. Its success gives us confidence that QCD is indeed the correct theory describing the strong nuclear interactions.

**Figure 3:**
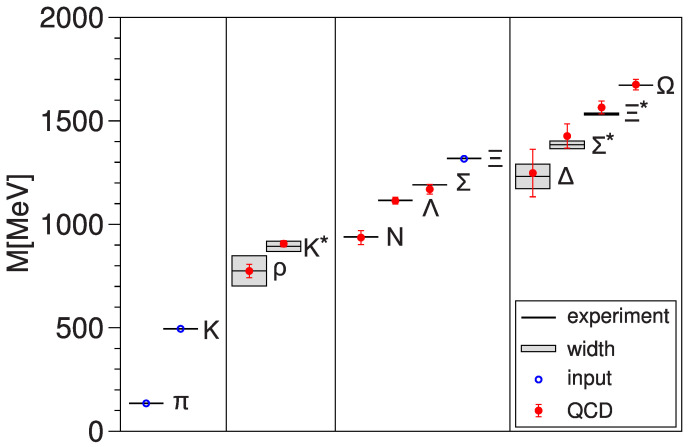
Hadron masses: comparison of theory and experiment. From [35].

I include a rather ancient plot (Figure 3) to illustrate this success.

There are many other successful lattice computations of experimentally measurable quantities. They include decay rates, quark masses and mixing matrix (CKM) elements. Many of these results can be found on the FLAG web site [36], which collects and critically evaluates them.

To sum up, LGT provides a very effective, nonperturbative way of comparing theory (for instance QCD) with experiment; so far, no significant discrepancies have been found.

## 8. Lattice Field Theory vs. Quantum Cellular Automata

Quantum Cellular Automata (QCA) [11,12,14,16] and LFTs can both be viewed as lattice versions of QFT. The main difference is that QCA are using a real-time evolution, which is unitary, whereas LFTs are using imaginary time steps. If all goes well, the two approaches should lead to the “same” continuum limit, meaning that the continuum limit of a LFT is the analytic continuation of the continuum limit of the corresponding QCA.

A minor point is that the QCA corresponding to a LFT containing bosons will require an infinite dimensional Hilbert space at each lattice point; since in the definition of a QCA it is often (but not always) assumed that each lattice point carries a finite dimensional space, this will then require a further approximation argument.

There is an alternative approach to LGT, which is even closer to QCA: the so-called Hamiltonian LGT proposed by Kogut and Susskind (KS-LGT) [37]. It defines a Hamiltonian on a spatial lattice; time is continuous. The theory can be seen as the limit of a QCA in which the time step $a_{t}$ has been taken to zero. Conversely, the discrete-time path integral for bosonic lattice field theory *is* a QCA [38] (albeit with an infinite dimensional Hilbert space at each lattice point).

One could let the unitary time evolution, exponentiating the Kogut–Susskind Hamiltonian, run for a finite time $a_{t}$ and so obtain a unitary evolution resembling a QCA. But, because this would involve infinitely many infinitesimal time steps, the evolution would be nonlocal, coupling infinitely many lattice points after the finite time step $a_{t}$. So, it would not be a QCA in the proper sense. Nevertheless, a proposal has been made to use the KS-LGT as a basis for a quantum simulation of the real-time evolution of a two-dimensional gauge theory [39].

Another difference between LFT on one side and QCA and Hamiltonian lattice field theory on the other is that in the latter time and space are treated differently, whereas in the former there is complete symmetry between time and space. The discretization in LGT breaks rotation invariance, whereas in QCA and KS-LGT it violates Lorentz invariance. In both approaches, it is necessary to take the continuum limit to have a chance to recover rotation invariance or Lorentz invariance, respectively. Conceivably QCA has a better chance for achieving this than KS-LGT because it already has a finite speed of propagation built into its formalism.

On the other hand, the Kogut–Susskind Hamiltonian (of course with a spatial cutoff in place) works directly in the appropriate Fock space for the gauge bosons and the fermionic matter fields; it does not require a somewhat elaborate construction like the one given in the QCA case for fermions [15,16], where at first a much larger Hilbert space of distinguishable particles is built, only to be reduced afterwards to the physical subspace.

QCA, as well as KS-LGT, require finding appropriate wave functions in the Fock space of the fields. A crucial requirement for a physical wave function in a gauge theory is the so-called Gauss constraint, which demands that the wave functions are independent of time-independent gauge transformations. The physical meaning of the constraint is the absence of immovable external charges, which in LGT is automatically assured by considering only gauge invariant functionals. Finding correct physical wave functions is difficult; even finding the correct vacuum wave function for an interacting QCA or a Hamiltonian model is a challenge, but it may be a rewarding task.

In LFT/LGT, on the other hand, one does not have to solve this problem: since

(73) $$\lim_{t\to \infty }\exp\left(-tH\right)=|0\rangle \langle 0|\;$$

as we have seen, the true ground state will emerge in the infinite volume limit automatically and is represented by the function 1 in the functional integral. On the other hand, finding the wave functions for the ground state, as well as other states in QCA or Hamiltonian LGT, might reveal physically interesting features. Some inspiration about how to approach this problem might be found in some older work on two-dimensional models in which the Dirac sea was “first drained and then refilled” [40,41].

## Data Availability

No new data were created or analyzed in this study.

## Appendix A. Derivation of Equation (66)

As mentioned, the Grassmann variables $\psi_{j},{\bar{\psi }}_{k}$ are nilpotent because they anticommute with themselves. In a finite volume, the number of linearly independent elements among these variables is finite. Call this number 2N, and then the power series

(A1) $$\exp\left(\bar{\psi },A\psi \right)=\sum_{n=1}^{\infty }\frac{1}{n!}{\left(\bar{\psi },A\psi \right)}^{n}$$

actually stops at the order *N*.

The Berezin integral over an element of the Grassmann algebra according to Equation (59) picks out the coefficient of the highest order term, so

(A2) $$\int \exp\left(\bar{\psi },A\psi \right)d\psi_{1}d{\bar{\psi }}_{1}\ldots d\psi_{N}d{\bar{\psi }}_{N}=\frac{1}{N!}\int {\left(\bar{\psi },A\psi \right)}^{N}d\psi_{1}d{\bar{\psi }}_{1}\ldots d\psi_{N}d{\bar{\psi }}_{N}$$

Note that

(A3) $$\left(\bar{\psi },A\psi \right)=\sum_{j,k=1}^{N}{\bar{\psi }}_{j}\psi_{k}A_{jk}\;,$$

(A2) still contains a sum over $N^{2N}$ terms, but most of them vanish because of the nilpotency. The surviving terms are the ones in which every $\psi_{j}$ and ${\bar{\psi }}_{k}$ appears exactly once. There are ${\left(N!\right)}^{2}$ of them because each possible permutation of the $\psi_{j}$, as well as the ${\bar{\psi }}_{j}$, appears.

By moving (commuting) bilinears ${\bar{\psi }}_{j}\psi_{k}A_{jk}$ around, we can always order the terms under the integral in such a way that the ${\bar{\psi }}_{j}$ appear in their natural order 1,…,N. That way, each term will appear N! times (from the permutations of the ${\bar{\psi }}_{j}$), so that N! different terms remain.

So, the surviving terms can be written as

(A4) $$\begin{array}{c} \sum_{p\in S_{N}}\epsilon_{p(1),\ldots ,p(N)}\int {\bar{\psi }}_{1}\psi_{p(1)}\ldots {\bar{\psi }}_{N}\psi_{p(N)}\epsilon_{p(1),\ldots ,p(N)}A_{1p(1)}\ldots A_{Np(N)}\;,\\ =\sum_{p\in S_{N}}\epsilon_{p(1),\ldots ,p(N)}A_{1p(1)}\ldots A_{Np(N)}\;, \end{array}$$

where *p* ranges over the group $S_{N}$ of permutations of the *N* elements and $\epsilon_{p(1),\ldots ,p(N)}$ is the sign of the permutation *p*, arising from moving the elements ${\bar{\psi }}_{n}$ into the standard order.

(A4) is just the definition of det(A).
